# Self-reported mental health in teenagers who received CI before the age of 2.5 years in relation to typical hearing peers and parents of both groups

**DOI:** 10.1371/journal.pone.0343241

**Published:** 2026-03-02

**Authors:** Anna Persson, Ulrika Löfkvist

**Affiliations:** 1 Department of Clinical Science, Intervention and Technology, Karolinska Institutet, Stockholm, Sweden; 2 Medical Unit Ear, Nose, Throat, Hearing and Balance, Karolinska University Hospital, Stockholm, Sweden; 3 Department of Public Health and Caring Sciences, Uppsala University, Uppsala, Sweden; Johns Hopkins School of Medicine and Kennedy Krieger Institute, UNITED STATES OF AMERICA

## Abstract

**Objective:**

The aim of this study was to investigate and compare how teenagers who received cochlear implants (CI) before 2.5 years of age perceive their own mental health, in relation to peers of the same age with typical hearing (TH). Comparisons were also made between teenagers’ self-reports and their parents’ assessments in both groups. Additionally, the study explored how various background factors might be associated with mental health outcomes.

**Materials and methods:**

The Strengths and Difficulties Questionnaire (SDQ), both the self-report and parent-report version, was used to assess the mental health of teenagers with CI (n = 26) and teenagers with TH (n = 57). SDQ total and subscale scores were compared between groups, between teenagers and their parents, and in relation to background characteristics collected via a questionnaire developed specifically for this study.

**Results:**

No significant differences in scores were found between teenagers with CI and TH. However, among those who reported clear difficulties, the challenges had persisted for a longer period of time for the CI group. Parents of the teenagers with CI scored similarly to their children on all scales except for Peer and Emotional problems. In contrast, parents of teenagers with TH scored significantly different from their children across all scales.

**Conclusion:**

The mental health outcomes by the SDQ suggest positive development trends in teenagers with CIs, when compared to findings from previous studies. These results indicate that early cochlear implantation, combined with family-centered habilitation, may establish essential conditions for improved quality of life and psychosocial well-being in this population.

## Introduction

In Sweden, pediatric cochlear implantation (CI) has been a treatment option for children born with severe to profound hearing loss, since the 1990s. Over the years, the age at which children receive their first CI has decreased dramatically [1]. Receiving CI at a young age has been found to be significantly associated with better hearing, speech and language development [1–3] Since approximately 95% of children with hearing loss have parents with typical hearing (TH) [4], and many have been full-time CI users from an early in life, it is reasonable to expect that their everyday life experiences differ from those of previous generations. However, little is known about the long-term effects of early implantation as these children become teenagers and young adults. Adolescents with CI is a heterogeneous group, partly due to different ages at 1^st^ CI but also in terms of individual and environmental factors. Individual factors may be related to etiology and comorbidities which may not only influence language, hearing and social skills, but also mental health.

Previous studies of children who received CI at later ages have reported that they experienced more mental health problems than peers with TH [5]. Children and teenagers with CI and/or hearing aids have also been found to show more depressive symptoms compared to age-matched peers with TH [6]. This is particularly concerning given that the adolescent years are important for children and young people’s continued opportunities for good and equal health throughout their lives. According to recent self-reported questionnaire data, over 50% of individuals aged 16–29 years with TH reported experiencing uneasiness, worry, or anxiety [7]. Also, depression affects young people somewhat more often than average. By the end of adolescence, 20% have been diagnosed with depression. Additionally, women are treated almost twice as often as men for anxiety, stress, and exhaustion syndrome [8].

This highlights the need for contemporary studies on the mental health of teenagers and young adults who received CIs at an early age, ideally including control groups with TH. However, there is currently a lack of data on long-term quality of life outcomes among Swedish children who received CI at an early age. To the authors’ knowledge, only two previous Swedish studies have investigated the mental health of children and adolescents who are deaf or hard of hearing in contexts similar to the current study [9,10]. In addition, two more studies have used SDQ to assess mental health in younger children with CIs: one involving children with incomplete partition type 3 (IP3) cochlear malformation [11], and another involving children with congenital cytomegalovirus (cCMV) infection [12]. Both studies included control groups with GJB2 mutations, a common non-syndormatic genetic cause of deafness. The findings showed that children with IP3 had poorer mental health outcomes, as reported by parents, compared to the control group [11], whereas no significant differences were found between the cCMV group and controls in parent-reported outcomes [12]. A notable limitation is the absence of self-reports from the children themselves and controls with TH.

Mejstad et al. [9] compared the results of a mental health questionnaire (Strength and Difficulties Questionnaire; SDQ) in pupils aged 11–18 years who were deaf and heard of hearing (n = 93). In short, the SDQ consists of 25 items rated on a three-point Likert scale that is dividided in five subscales; Emotional problems, Conduct problems, Hyperactivity/inattention, Peer problems and Prosocial behavior (further details on the SDQ are provided in the Methods section). The results from Mejstad et al. [9] were presented by school setting, including mainstream schools (n = 50), schools for the hard of hearing (n = 19), and schools for the deaf (n = 24). Students in the schools for the deaf reported the lowest scores across all subscales compared to students in the other school settings. Teenagers who used sign language as their primary mode of communication rated their mental health lower than those in the other groups [9]. Important to note is that as this study was conducted before CIs were routinely provided at an early age and therefore included only three participants using cochlear implants [9]. However, the association between schooling and mental health problems in adolescents with CIs has been confirmed in other studies. Huber et al. [13] found that students attending special schools had significantly higher total difficulty scores on the SDQ (i.e., worse), particularly regarding conduct problems, compared to students in mainstream schools. Possible explanatory factors included hearing-related variables, like age at implantation of 2nd CI (in cases of bilateral implantation), understanding of speech in noise, as well as educational background, sign language use and competence, and the children’s social background [14].

Anmyr et al. [10] compared SDQ self-reports, alongside the parental version (SDQ-P) and teacher version (SDQ-T). The study included 22 participants aged 9–15 years, all of whom had received cochlear implants at a mean age of 4.6 years (SD = 2.0). At the time of testing, the participants had a mean age of 6.9 years of CI experience (SD = 3.0). The results showed that almost a quarter of the children had total scores indicating poor mental health, with 9% classified as borderline and 14% as abnormal according to normative data [10]. Furthermore, the younger age group (9–12 years, n = 12) scored significantly higher (i.e., worse) on emotional symptoms compared to the older age group (12–15 years, n = 10). There were no differences between parent and teacher ratings for the same child, and the authors concluded that the children with CIs reported greater difficulties than were observed by their parents and teachers. Socio-demografic characteristics, hearing-related factors, and speech and language development showed no significant correlations with mental health outcomes. Besides improvements over time in children with severe to profound hearing loss, neither Mejstad et al. [9] nor Anmyr et al. [10] included control groups with typically hearing children.

In Norway, Haukedal and colleagues examined health-related quailty of life (HRQoL) in children with CI and controls with TH, aged 5;5–12;11 years [15]. The results from parent-reports showed that about 50% of the children with CI had HRQoL levels comparable to those of controls with TH. Their findings also confirmed an association between HRQoL and better language skills [15]. In a later study, Haukedal et al. [16] found that children aged 5;6–13;1 years with CI reported somewhat lower self-perceived HRQoL compared to controls with TH. Specific areas of difficulty included social interactions and managing school-related challenges. Futhermore, an age effect was observed, with older children reporting better outcomes. Another finding was a sex effect, with more girls reporting emotional distress, while boys instead had more difficulties with behaviour issues [16]. In contrast to the heterogeneous population of children with hearing loss, this cohort did not include individuals with additional diagnoses or a multilingual background.

A recent study using the SDQ to evaluate quality of life in Norwegian deaf and hard of hearing (DHoH) youth found that parents reported significantly more problems, particularly in the peer domain, compared to parents of youth with TH [17]. Self-reported total SDQ scores did not differ between DHoH adolescents and their TH peers, albeit DHoH youth scored higher (i.e., worse) on Emotional and Peer problems and lower on Conduct problems [17]. Additionally, adolescent girls reported better prosocial behavior but significantly more problems with emotional difficulties than boys.

Historically, the majority of children in Sweden who received CIs during the first decade were enrolled in special education pre-/schools that promoted the development of both spoken and sign language. These educational settings were limited in number, meaning that many children had to travel by school taxi. In Sweden, most children have started preschool at 18 months of age. Expectations for spoken language development in children with CI at that time were generally very low. It was not until 2003 that intervention options focusing on learning spoken language through listening were introduced for Swedish children and their parents [18]. Over time, a shift occurred, with children who received CIs early increasingly attending mainstream pre-/schools closer to their home. In terms of generational change, Hammer et al. [19] concluded that it is now time to raise expectations for children born with hearing loss and provided with hearing technology before the age of one year – both regarding auditory and spoken language outcomes and overall well-being [19]. In their study of 22 Danish children aged 9–12 years, 73% of whom had received CIs at a mean age of 16.27 months, Hammer et al found high self-reported scores on the SDQ (i.e., positive) [19]. A possible bias in these results was that all participating children and their parents had undergone intensive educational training in Auditory-Verbal Therapy, and children with additional diagnoses were excluded from the study [19].

Older school-aged children and adolescents are in transitional phases of development, moving from childhood through adolescence into adulthood. The risk of depression and other mental health problems increases dramatically during adolescence, and therefore the prevention of depression during this period is especially prioritized for all adolescents [20,21]. Little is yet known about how CI-users handle these developmental stages, while simultaneously adjusting their listening abilities with CIs, often in noisy environments and with less support from others [22]. Higher-level cognitive abilities related to executive functions continue to develop and are not fully mature until around 20–25 years of age [23]. Early identification of mental health issues may therefore allow targeted interventions for individuals who receive CI at a very early age. Only recently has the first cohort of children in Sweden who received CIs before the age of 2.5 years reached adolescence at a group level. This motivates longitudinal studies of mental health in teenagers with CIs, to examine and determine the causal factors contributing to higher levels of mental health symptoms.

The aim of this study was to investigate how a cohort of teenagers who received CIs before the age of 2.5 years perceive their own mental health. These self-reports were compared both to peers of the same age with TH and to their parents’ assessments. The following reserach questions were posed:

- Do teenagers who received a CI before the age of 2.5 years report similar levels and patterns of mental health as age-matched peers with typical hearing?
- Do teenagers with CI and those with TH show similar levels of agreement with their parents’ ratings of mental health?
- Which background factors – such as sex, age at first CI, etiology, languages spoken at home, parental education level, and absence of additional disabilities – are associated with mental health outcomes in teenagers with CI and TH?

## Materials and methods

Ethical approval for this study was granted by the Swedish Ethical Review Authority (Umeå avdelning medicin) with Dnr 2021-04345. The start of the recruitment for this study was 10/01/2022 and it ended 10/06/2024. According to Swedish ethics, children above the age of 6 years must be given written information at level appropriate to their age and teenagers above the age of 13 years must approve and give their own consent, despite the decision of their parents. For this study, age-appropriate information about the study for the teenagers was written and approved by the Ethical committée. All teenagers and parents needed to sign an informed consent (paper version) to be included in this study. For teenagers with CI and their parents, information about the study was given in both writing and verbally. The teenagers with typical hearing and their parents received the information about the study in writing. Teenagers and parents in both groups were given separate envelopes to return their questionnaires independently.

### Participants

Participants with CIs were invited to take part in the study if they had received their first CI before the age of 2.5 years, were between 12–17 years and followed the mainstream curriculum, in a school were Swedish was the language of instruction. To ensure that the participants had the possibility of developing spoken language, individuals that had not been full-time users of their CIs during childhood were not invited. Furthermore, at least one parent needed to speak Swedish. As neurodevelopmental conditions are common in the population of hearing loss at large, careful background data was collected for children with comorbid conditions as this has been found to impact overall development. The inclusionary criteria were the same for participants and their parents in the control group, albeit they needed to have typical hearing.

The current study group was part of a cross-professional study, recently described in Löfkvist et al. [18]; The TeenAgers and Young Adults with Cochlear Implants study (TAYACI). The TAYACI study includes participants between the ages of 12–22 years, of which all had received their first CI before 2.5 years of age. In addition to mental health, assessments were also made in the areas of hearing, balance, language, and etiology. Participants with CI were recruited from the Hearing Implant Center (HIC) at Karolinska University Hospital where all came for regular clinical check-ups. Eligible participants were sent an information letter about the study by mail. A reminder letter was sent after a month to those who had not responded. A total of 108 study invitations were sent and of those; 53 accepted the invitation; 11 declined to participate; and the remaining 44 did not respond. Out of the total 53 participants, 27 were between 12–17 years of age. The control group were recruited through flyers, social media and snowball sampling. Interested eligible participants with TH were invited to perform the same test battery as the participants with CI but could also choose to take part in certain sub studies within the larger project (described in Löfkvist et al., 2025) [18]. Those who came in person for additional assessments on site were given the SDQ and background data in paper format to be returned the same day. In cases where the participant had not completed the questionnaires on site, they were given a stamped envelope to return the papers via mail. For those who enrolled in the study for questionnaires only, paper versions were sent home via mail including a stamped envelope to return the data. The demographic information of the final participants for the current study can be found in Table 1.

**Table 1:** Demographic characteristics of study participants. Background variables in the study participants with cochlear implants (n = 26) and typical hearing (n = 57).

Variable	Teenagers with CI (n = 26)	Teenagers with TH (n = 57)
Chronological age (years)	15.3 (1.5), range: 12.7–17.7	15.0 (1.6), 11.7-17.9
Sex	Females (n = 14) Males (n = 12)	Females (n = 32) Males (n = 23) Non-binary (n = 2)
Age at first CI surgery (months)	14.5 (6.2), range: 7.3–26.7	N/A
Cochlear implants	Bilateral (n = 23) Unilateral, no hearing aid (n = 3)	N/A
Etiology	Unknown (n = 8) Non-syndromic genetic (n = 5) Syndromic genetic (n = 6) Congenital or prelingually acquired cause (n = 7)	N/A
Additional diagnoses	ADHD and Autism (n = 2) ADHD and Dyslexia (n = 1) DLD* and Dyslexia (n = 1) Dyslexia (n = 2)	ADHD (n = 2) Autism (n = 2) Dyslexia (n = 3)
Communication modality (selfreported)	Spoken Swedish (n = 21) Spoken Swedish + other spoken languages (n = 2) Spoken Swedish + sign language (n = 2) Spoken Swedish + sign supported Swedish (n = 1)	Spoken Swedish (n = 46) Spoken Swedish + other spoken languages (n = 11)
Parental education level > university level	Mothers (n = 13) Fathers (n = 13)	Mothers (n = 42) Fathers (n = 40)
Educational placement	Preschool:Mainstream (n = 25), deaf group (n = 1) Elementary: Mainstream (n = 20), mainstream and hard of hearing class (n = 5), hard of hearing class (n = 1) High school: Mainstream (n = 9), highschool for hard of hearing (n = 1)	Preschool: Mainstream (n = 56), special group (n = 1) Elementary: Mainstream (n = 56), special school (n = 1) High school: Mainstream (n = 19), special high school (n = 1)

### Materials

For this study, we used the the Swedish versions of the Strengths and Difficulties Questionnaire version for the ages 4–17 (SDQ 4–17) [24,25] and the parent-version (SDQ-P) [25,26]. The SDQ has been found to be a reliable and valid questionnaire for use with DHoH children [5,10,12,27]. It contains 25 statements addressing four areas of difficulties and one area of strength, along with questions about the impact of these difficulties on the individual. Higher scores on the four areas of difficulties indicates worse mental health, with reversed scoring on the area of strentgh. Each version of the SDQ takes around 5–10 minutes to complete. In addition, a background questionnaire specifically designed for this study was used to gather information on neurodevelopmental conditions and other background factors hypothesized to influence mental health.

### Statistical analyses

Data are presented as means and standard deviations to facilitate comparisons between the groups included in this study and with previous studies using the SDQ. To compare differences in scores between teenagers and their parents, we used the nonparametric one-way Mann-Whitney U test. Pearson’s correlation coefficients were calculated to examine associations between background variables and study outcomes. All analyses were conducted using SPSS software (version 28). The significance level was set at *p* < .05.

## Results

**Fig 1:**
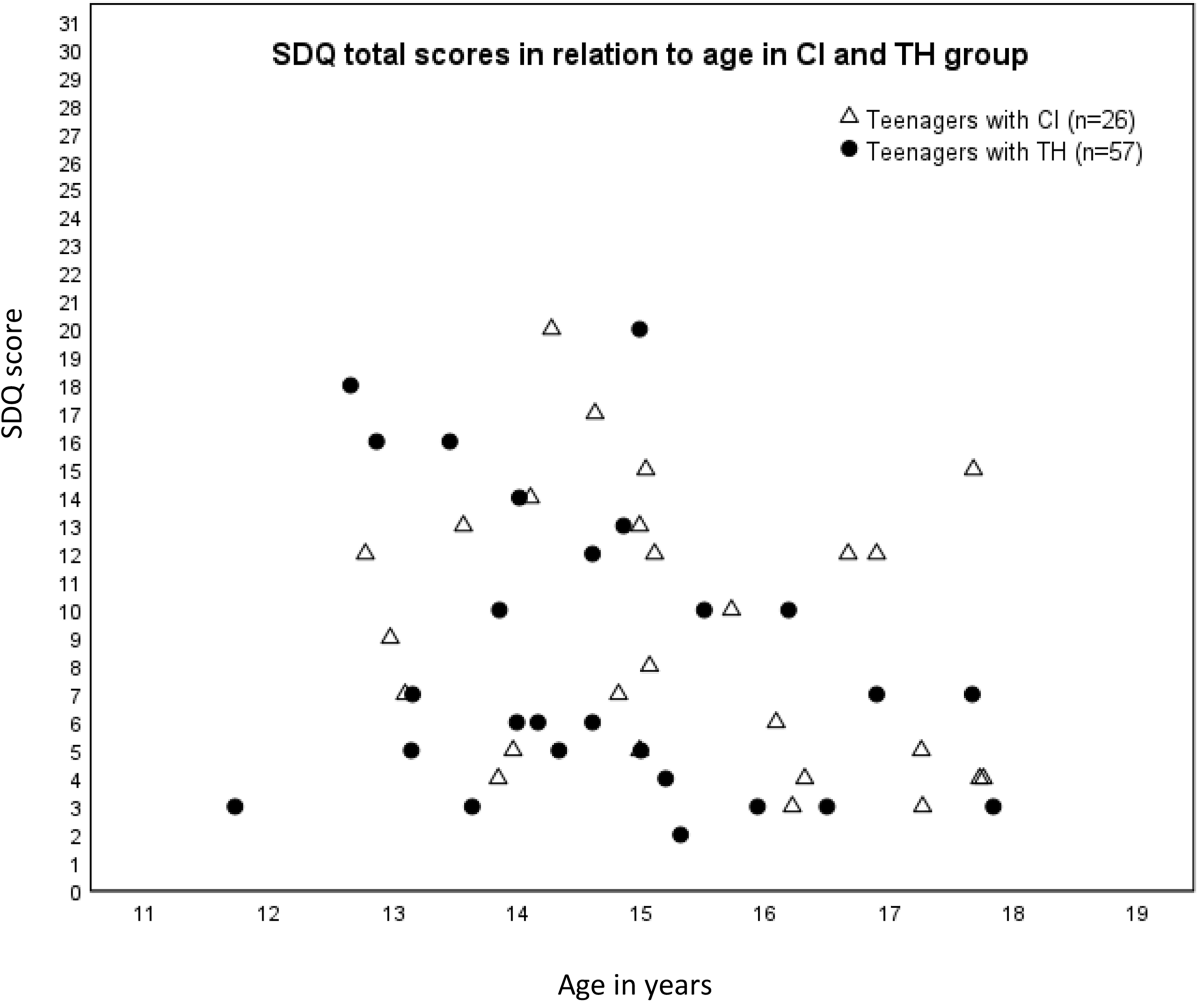
SDQ total scores in relation to age and study group. Total SDQ scores in individual teenagers with cochlear implants (n = 26) and typical hearing (n = 57). Score scale 0-40: *normal* 0-15, *borderline* 16-19, *abnormal*, 20-40.

Descriptive distributions of both groups are illustrated in Fig 1. Although the mean scores of teenagers with CI were slightly higher than those of the TH group, no statistically significant differences were found between the two groups on any of the SDQ subscales. Furthermore, no significant differences were found between sexes, either within or between the groups of CI and TH (*p’s* > .05).

The mean scores on all SDQ scales of both groups of teenagers and their parents are found in Table 2. At a group level, the scores on all scales showed similar numbers of the teenagers with CI and their parents on. There was a statistically significant difference on the Emotional scale (Emotional symptoms: Mann-Whitney U = Z = −3, 269, *p* = .001) and Peer problems (Mann-Whitney U = Z = −2,402, *p* = .016). The teenagers with TH and their parents scored statistically significantly different on all scales except the Prosocial behavior scale (Total difficulties: Mann-Whitney U = Z = −4,606, *p* = < ,001; Emotional symptoms: Mann-Whitney U = Z = −4, 315, *p* = < .001; Hyperactivity-Inattention: Mann-Whitney U = Z = −3,964, *p* = < .001; Conduct problems: Mann-Whitney U = Z = −3,090, *p* = < .001; Peer problems: Mann-Whitney U = Z = −2,225, *p* = .026).

**Table 2:** Total SDQ and subscales scores. Teenagers mental health SDQ in total difficulties and subscales, distributed by self-report and parental report for experimental and control group; mean, S.D., min-max.

Total SDQ and subscales	Self-report CI (n = 26)	Self-report TH (n = 47)	Parents CI (n = 26)	Parents TH (n = 47)
Total difficulties^a^	9.2 (4.8) 3–20	8.4 (5.2) 2–26	8.8 (5.7) 0–17	4.3 (3.5) 0–16
Emotional symptoms^b^	2.6 (2.0) 0–7	2.5 (2.0) 0–8	1.1 (1.9) 0–6	1.2 (1.5) 0–7
Hyperactivity-inattention^b^	3.1 (1.9) 0–7	2.3 (2.3) 0–8	3.0 (2.1) 0–7	1.7 (1.8) 0–7
Conduct problems^b^	1.5 (1.7) 0–6	1.2 (1.5) 0–5	1.4 (1.3) 0–4	0.5 (1.0) 0–4
Peer problems^b^	1.9 (1.5) 0–5	1.4 (1.4) 0–6	3.3 (2.2) 0–7	0.9 (1.1) 0–4
Prosocial behavior^b^	8.8 (1.4) 5–10	8.7 (1.5) 5–10	9.0 (1.6) 5–10	8.9 (2.0) 3–18

^a^Possible range of score is 0–40. Note that the prosocial subscale does not contribute to the total sum.

^b^Possible range of score is 0–10. Note that the prosocial scale is scored in reverse so that lower scores indicates difficulties.

In terms of background factors for teenagers with CI, chronological age showed a moderate correlation that was significant to the Conduct scale (*r* = .43, *p* = 0.005) but not any of the other scales. When the CI group was divided into two age brackets (12−14 years and 15−17 years), the correlation was further confirmed by a significant difference between the younger and older teenagers (Mann-Whitney U = Z = −2.271, *p* = 0.023) a. Specifically, the younger group scored higher (M = 2.23, SD 1.78) compared to the older group (M = 0.84, SD 1.34).

Age at first cochlear implantation did not show statistically significant correlations with any of the SDQ scales. Similarly, the presence of additional disabilities was not significantly correlated with SDQ outcomes. However, it is worth noting that among the two participants with CI with total SDQ scores in the abnormal range, one had a diagnosis of both ADHD and autism-spectrum disorder, while the other had siblings diagnosed with ADHD. Other background factors like etiology, languages spoken at home, and parental education level did not show any significant correlations with any of the SDQ scales.

The number of teenagers and their parents who scored within the borderline or abnormal ranges is presented in Table 3. The percentage of teenagers with CI and those with TH indicating signs of mental ill-health ranged from 4% to 18% across the various SDQ scales. Among teenagers with CI, the highest rates were observed in the Conduct problems and Peer problems scales, whereas for the TH group, the highest levels were reported on the Hyperactivity-Inattention scale. Notebly, more than half (54%) of the parents of teenagers with CI perceived their child to have Peer problems. In contrast, parents of teenagers with TH reported fewer problems than their children on all scales, except for the Emotional Symtoms scale.

**Table 3:** Borderline/abnormal scores on SDQ. Number and percentage of participants with borderline/abnormal scores on the SDQ.

Total SDQ and subscales	Teenagers CI (n = 26)	Teenagers TH (n = 57)	Cut-off	Parents CI (n = 26)	Parents TH (n = 57)	Cut-off
Total difficulties, *n* (%)	2 (8)	6 (11)	16	3 (12)	0 (0)	17
Emotional symptoms, *n* (%)	2 (8)	6 (11)	6	2 (8)	2 (8)	6
Hyperactivity-inattention, *n* (%)	3 (12)	10 (18)	6	3 (12)	1 (2)	7
Conduct problems, *n* (%)	4 (15)	5 (9)	4	0 (0)	0 (0)	5
Peer problems, *n* (%)	4 (15)	5 (9)	4	14 (54)	2 (8)	4
Prosocial behavior, *n* (%)	1 (4)	2 (4)	5	1 (4)	2 (8)	5

*Note:* CI = cochlear implants; TH = typical hearing. The Prosocial subscale is not included in the total difficulties score. Lower scores on the Prosocial scale indicate greater difficulties.

When comparing the SDQ scores of the current group of teenagers with CI to those reported in previous Swedish studies, the results suggest similar or lower scores, indicating better mental health outcomes (see Table 4). Based on face validity, their mean group scores most closely resemble those of hard-of-hearing participants attending mainstream schools, as reported by Mejstad et al. [9].

**Table 4:** Current versus previous SDQ scores. Results on SDQ of current teenagers with CI (TACI) compared to previous studies (mean and SD).

Total SDQ and subscales	TACI (n = 26)	Anmyr (2012) School for DHoH (n = 22)	Mejstad (2009) Schools for the deaf (n = 24)	Mejstad (2009) Mainstream (n = 50)
	Mean (SD)	Mean (SD)	Mean (SD)	Mean (SD)
Total difficulties^a^	9.2 (4.8)	11.5 (5.9)	11.9 (5.1)	8.8 (5.9)
Emotional symptoms^b^	2.6 (2.0)	3.5 (2.3)	3.5 (2.7)	2.6 (2.1)
Hyperactivity-inattention^b^	3.1 (1.9)	3.6 (2.2)	3.3 (1.8)	3.1 (2.2)
Conduct problems^b^	1.5 (1.7)	2.0 (1.8)	2.5 (1.5)	1.2 (1.6)
Peer problems^b^	1.9 (1.5)	2.3 (1.5)	2.7 (1.8)	1.9 (2.0)
Prosocial behavior^b^	8.8 (1.4)	8.0 (1.6)	7.0 (1.6)	9.0 (1.6)

^a^Possible range of score is 0–40. Note that the prosocial subscale does not contribute to the total sum.

^b^Possible range of score is 0–10. Note that lower score on the prosocial scale indicates difficulties.

Self- and parent-reported difficulties related to emotions, concentration, conduct, getting along and engaging with other people are presented in Table 5. A total of 58% of the teenagers with CI and 42% of those with TH reported experiencing minor to significant difficulties. More than half of the parents of teenagers with CI reported that their child had no difficulties, while 80% of parents in the TH group reported the same. Teenagers with CI most frequently reported difficulties in school and with friends, and their parents reported the same areas, with peer relationships being of particular concern. Teenagers with TH also reported school and friends as key areas of difficulty but additionally noted challenges during leisure time. Regarding duration, most teenagers with CI and their parents reported that the difficulties had persisted for over a year, whereas reports from the TH group indicated a shorter and more variable time span. Across both groups, parents were more likely than their children to report that the difficulties had a greater impact on others around them.

**Table 5:** SDQ scores, part-II. Self- and parent-reported perceived difficulties based on SDQ Part II among teenagers with CI, teenagers with TH, and their parents. Values are presented as number and percentage; *n* (%).

*Do you think that you/your child has experienced difficulties with any of the following: emotions, concentration, behavior, or getting along and socializing with other people?*	No	Yes, small difficulties	Yes, clear difficulties	Yes, severe difficulties
Teenagers with CI (n = 26)	11 (42)	12 (46)	2 (12)	0 (0)
Teenagers with TH (n = 46)	26 (56)	15 (32)	5 (10)	1 (2)
Parents of CI (n = 26)	16 (62)	6 (23)	4 (15)	0 (0)
Parents of TH (n = 46)	37 (80)	8 (17)	3 (7)	0 (0)
*If yes, for how long has the difficulties been present?*	< 1 month	1-5 months	6-12 months	>1 year
Teenagers with CI (n = 14)	0 (0)	2 (14)	1 (7)	11 (79)
Teenagers with TH (n = 19)	0 (0)	4 (21)	13 (68)	2 (11)
Parents of CI (n = 10)	0 (0)	0 (0)	1 (10)	9 (90)
Parents of TH (n = 11)	0 (0)	1 (9)	6 (55)	4 (36)
*Are you bothered or worried by the difficulties?*	Not at all	Just a little	Quite a lot	Very much
Teenagers with CI (n = 14)	0 (0)	10 (71)	4 (29)	0 (0)
Teenagers with TH (n = 19)	0 (0)	14 (74)	4 (21)	1 (5)
Parents of CI (n = 10)	0 (0)	7 (70)	3 (30)	0 (0)
Parents of TH (n = 11)	0 (0)	4 (36)	5 (45)	2 (19)
*Do the difficulties bother you in the following areas?* *a) At home/with your family* *b) With friends* *c) In school* *d) Leisure time*	Not at all	Just a little	Quite a lot	Very much
Teenagers with CI (n = 13)	a) 0 (0)	a) 11 (85)	a) 2 (15)	a) 0 (0)
	b) 0 (0)	b) 9 (69)	b) 3 (23)	b) 1 (8)
	c) 0 (0)	c) 5 (38)	c) 7 (54)	c) 1 (8)
	d) 13 (100)	d) 0 (0)	d) 0 (0)	d) 0 (0)
Teenagers with TH (n = 19)	a) 14 (74)	a) 3 (16)	a) 2 (10)	a) 0 (0)
	b) 11 (58)	b) 4 (21)	b) 3 (16)	b) 1 (5)
	c) 7 (37)	c) 5 (26)	c) 5 (26)	c) 2 (11)
	d) 12 (63)	d) 4 (21)	d) 3 (16)	d) 0 (0)
Parents of teenagers with CI (n = 10)	a) 0 (0)	a) 6 (60)	a) 4 (40)	a) 0 (0)
	b) 0 (0)	b) 9 (90)	b) 1 (10)	b) 0 (0)
	c) 0 (0)	c) 6 (60)	c) 3 (30)	c) 1 (10)
	d) 0 (0)	d) 9 (90)	d) 1 (10)	d) 0 (0)
Parents of teenagers with TH (n = 11)	a) 5 (45)	a) 3 (27)	a) 3 (28)	a) 0 (0)
	b) 4 (36)	b) 7 (64)	b) 0 (0)	b) 0 (0)
	c) 4 (36)	c) 6 (54)	c) 1 (9)	c) 0 (0)
	d) 5 (45)	d) 5 (45)	d) 1 (10)	d) 0 (0)
*Do you think that the difficulties are hard on the people around you (family, friends, etc.)?*	Not at all	Just a little	Quite a lot	Very much
Teenagers with CI (n = 14)	5 (36)	5 (36)	4 (28)	0 (0)
Teenagers with TH (n = 19)	0 (0)	14 (74)	4 (21)	1 (5)
Parents of CI (n = 10)	0 (0)	6 (60)	4 (40)	0 (0)
Parents of TH (n = 11)	0 (0)	4 (36)	4 (36)	3 (28)

*Note:* CI = cochlear implant; TH = typical hearing. On the Prosocial subscale, lower scores indicate greater difficulties. The percentages may not total 100% due to rounding.

## Discussion

At a group level, teenagers with CI showed similar overall results on the SDQ as their peers with TH. However, a slightly higher proportion of teenagers with CI fell within the borderline or abnormal range on the Conduct problems and Peer problems subscales, consistent with findings from Hammer et al. [19]. Furthermore, a greater number of teenagers with CI reported that their difficulties had persisted for over a year. These challenges may be related to early-onset factors such as deafness and/or co-occurring developmental conditions; however, situational or recently emerging factors (e.g., increased academic demands, changes in family constellation, etc.) cannot be excluded.

The groups were largely similar in terms of schooling and home language, which may have contributed to comparable SDQ outcomes. All participants with CI received their technology at an early age and all (except one participant in the CI group) attended mainstream school settings from preschool and had spoken Swedish as their primary communication mode. There was a similar number of teenagers with additional disabilities and variations in spoken languages at home in both groups. A large proportion of the parents in both groups were highly educated, which may have affected outcomes to a similar extent in both groups.

In contrast to previous studies, our findings suggest that teenagers with CI report somewhat less difficulties than their parents – an inverse pattern compared to Anmyr et al. [10]. Notably, discrepancies between self- and parent-reports in the CI group were observed only on the Emotional and Peer Problems scales, with otherwise similar scores across the remaining SDQ domains. Interestingly, teenagers with TH scored signficantly different compared to their parents to a much larger extent. One possible explanation is that parents of adolescents with CI may be more attuned to emotional and peer-related functioning due to their child’s medical history and potential vulnerability. Many participants with CI and their parents in this study had received intensive early family-centered intervention such as Auditory-Verbal Therapy, which may also have contributed to parental awareness and responsiveness; however, this was not directly tested in the present study.

Importantly, the intervention options available to the participants in the current study were not accessible at the time of earlier research [9]. Similar to findings in the Norwegian study by Overgaard et al. [17], parents in the present study reported higher scores than their children on the Peer problems scale. It is not surprising that peer-related challenges is something that parents worry about as friendship quality has been found to be positively associated with subjective well-being [28]. As children grow older, organizing social acitivites increasingly moves beyond parental influence. Considering the ongoing digital evolution, one possible explanation for the elevated Peer problems scores (reported by parents) is a shift from in-person socializing to digital communication. All teenagers in this study reported having their first exposure to screens between the ages of 0 and 8 years. Daily screen time ranged from a minimum of one hour to more than five hours per day, with over 60% of the teenagers reporting screen use exceeding five hours daily. The differences in scores between teenagers and parents on Emotional problems were found in both groups. This could to some extent be explained by the nature of being a teenager which constitutes a period of growth in life with challenges and social role changes they may not want to share with their parents.

One limitation of this study is the relatively small sample size, particularly in the CI group. This may have reduced statistical power, especially given that outcomes were further divided across SDQ subscales, thereby limiting the ability to detect small group differences. Although similar sample sizes and subscale-based SDQ analyses have been reported in previous studies, the present findings should nevertheless be interpreted with caution. In addition, unequal group sizes between adolescents with CI and those with typical hearing may have further affected statistical power in between-group comparisons. The choice of Pearson’s correlation over Spearman’s was under consideration, as some variables showed non-normal distributions. This was to some point expected considering the inclusion of adolescents with additional disabilities. However, analyses did not affect the significance of the results in either direction. We further acknowledge that the results may have been affected due to the discrepancy of participants in the CI and TH groups. The reason to why we decided to include more subjects with TH in our study was based on that research on groups of children with HL often show larger outcome variances compared to groups with TH. Another limitation concerns recruitment pathways. Adolescents with CI were recruited via a tertiary clinical center, whereas adolescents with typical hearing were recruited through social media and snowball sampling. Although no group differences were observed in reported screen time or key background characteristics, differences related to recruitment methods may have influenced sample composition. This potential selection bias should therefore be considered when interpreting the findings.

This study is one of the few to investigate mental health in teenagers with CI, comparing them with age-matched peers with TH, as well as with parents from both groups. The sample is unique in the sense that all participants with CIs received their first implant before the age of 2.5 years and were full-time users of their devices, which provide us with a contemporary view on what it is like being a teenager with CI in Sweden today. The study also included multilingual participants and teenagers with additional disabilities. Although no statistically significant group-level differences in mental health outcomes were observed based on these factors, borderline or abnormal results were reported among participants with comorbid conditions. Comorbidity is known to affect well-being negatively as the conditions often reinforce each other, which leads to poorer health development, reduced quality of life, increased suffering and greater risk of social problems such as isolation. Therefore, it is possible that the results of these participants may have confounded the results. It also indicates that children with CI and other disabilities should receive specific attention in the intervention process.

In line with Hammer et al. [19], our results indicate that we can raise our expectations for children born with hearing loss who receive early hearing technology in terms of overall well-being. However, it remains essential to acknowledge individual conditions and contextual factors. Given that hearing loss is a lifelong condition and that mental health state may change over time, it appears warranted to include systematic intervention with a focus on mental health support as part of early habilitation programs, regardless of age at cochlear implantation.

In sum, the outcomes on mental health, as measured by the SDQ, indicate a positive development among teenagers with CIs compared to findings in previous studies [9]. The results suggest that early intervention through cochlear implantation together with early family-centered habilitation provides these children with fundamental conditions that support long-term improvements in quality of life. The next phase of the TAYACI study will involve analyzing results from young adults using the same instruments, to explore how the transition to adulthood may impact mental health. Further extensions of these results will include analyses of associations between mental health outcomes and collected data on participants’ language skills. Future studies should aim to include larger and more diverse samples of individuals with CI from different regions of Sweden and should specifically examine mental health among adolescents who received their cochlear implants before 12 months of age.
